# Factors Affecting Levels of Airborne Bacteria in Dairy Farms: A Review

**DOI:** 10.3390/ani10030526

**Published:** 2020-03-21

**Authors:** Álvaro Rafael Quintana, Susana Seseña, Ana Garzón, Ramón Arias

**Affiliations:** 1 Instituto Regional de Investigación y Desarrollo Agroalimentario y Forestal de Castilla La Mancha (IRIAF), 13300 CERSYRA de Valdepeñas (Ciudad Real), Spain; rarias@jccm.es; 2 Departamento de Química Analítica y Tecnología de los Alimentos, Facultad de Ciencias Ambientales y Bioquímica, Universidad de Castilla-La Mancha, 45071 Toledo, Spain; Susana.SPrieto@uclm.es; 3 Departamento de Producción Animal, Universidad de Córdoba, 14071 Córdoba, Spain; pa1gasia@uco.es

**Keywords:** dairy farm environment, air quality, airborne microorganism, milk

## Abstract

**Simple Summary:**

The environmental quality of farms plays an important role in the food safety of the dairy industry because it may influence the microbial communities in milk. The microorganisms present in the different areas of a farm have an influence on this environmental quality, using the air as a vehicle of dissemination. However, the ability of this airborne microbial community to contaminate the milk, like the sources of origin of these microorganisms, has not been well studied in dairy farms until now. This review examines the importance of different factors that affect the environmental quality of dairy farms, and must, therefore, be taken into account when considering the importance of environmental microbiology as a tool in the improvement of the quality of milk and dairy products.

**Abstract:**

This review attempts to reflect the importance of different factors that affect the environmental quality of dairy farms and must, therefore, be taken into account when considering the importance of environmental microbiology as a tool in the improvement of the quality of milk and dairy products. The effect of a factor such as temperature is vital for the dairy farm environment, especially when the temperatures are extreme, because a proper choice of temperature range improves the quality of the air and, thus, animal welfare. Similarly, the appropriate level of relative humidity in the environment should be taken into consideration to avoid the proliferation of microorganisms on the farm. Air quality, well-designed livestock housing, proper hygienic practices on the farm, stocking density, and the materials used in the livestock houses are all important factors in the concentration of microorganisms in the environment, promoting better welfare for the animals. In addition, a ventilation system is required to prevent the pollution of the farm environment. It is demonstrated that proper ventilation reduces the microbial load of the environment of dairy farms, enhancing the quality of the air and, therefore, the wellbeing of the animals. All this information is very useful to establish certain standards on dairy farms to improve the quality of the environment and, thereby, achieve better quality milk and dairy products.

## 1. Introduction

Due to the beneficial properties of milk and the wide range of essential nutrients it provides, there are more than 6 billion consumers of milk and milk products throughout the world, the majority of them in developing countries [1]. Throughout the twentieth century, milk became a raw material of an important industry and was made available to consumers in a comfortable, safe, and economic way. Nowadays, milk production is a dynamic and growing industry that is essential to the economies of many countries. Approximately 150 million family farms around the globe are engaged in milk production [2]. The farmgate milk price depends on the milk’s compositional quality, but the hygienic quality of the milk is also very important.

The control of milk quality is one of the most important problems in the dairy sector. Although traditionally the quality of the milk was evaluated according to its composition, due to the high levels of milk production at the end of the last century, quality control systems are now based on health and hygienic parameters too, specifically on the total mesophilic bacteria and the somatic cell count in milk from bulk tanks [3]. Laws on food security and nutrition, such as Regulation EC n° 853/2004 [4] in Europe, usually establish the boundaries for these parameters.

The presence of microorganisms in milk can have their origin in intramammary infections (affecting somatic cell count) and/or environmental contamination (affecting total aerobic microbial count), such as udder hygiene, milking parlor, milk houses, etc. [5]. Gonzalo et al. [6] pointed out that the total bacteria counts allow the evaluation of the level of adequacy of cleaning practices on the dairy farm, in addition to ensuring the correct quality of the milk and milk products that derive from it. However, this count is less representative for the evaluation of the microbial quality of milk, and that is why several studies have assessed other bacterial groups of milk in cattle [7], in goats [8], and in sheep [9].

Milk microbiota has a great influence on the safety and quality of dairy products, carrying out various functions such as facilitating dairy fermentations or causing spoilage or disease, among others [10]. For this reason, it becomes very important to know the variety of routes that these microorganisms could use to reach the milk. Good veterinary practices can be implemented, as well as good management and hygiene of the milking parlor, which can avoid direct contamination of the milk [11]. However, it is not so easy to control the environmental quality of dairy farms.

In recent years, the dairy farm environment has become a focus of interest in the agro-food industry. This is due to the effects of the diversity of activities carried out on the farms, which could affect the final product, the milk, and, therefore, could have social and economic impacts on the production of both milk and dairy products (mainly cheese). Regarding the importance of environmental microbiology on dairy farms and on the quality of milk and dairy products, both airborne bacteria and the main factors that affect the microbial load of the dairy farm environment will be examined in this review to recommend adequate preventive measures on the farm.

## 2. Methodology

The review includes primarily references published in journals cited in the ScienceDirect database (https://www.sciencedirect.com/); papers published in these journals have been refereed. Various search terms have been employed to identify relevant publications (e.g., ‘air quality’, ‘airborne microorganism’, ‘environment’, ‘dairy farm’, ‘air contamination’, ‘milk’, ‘factors’, ‘welfare’, ‘hygiene’, ’humidity’, ‘temperature’, ‘ventilation effects’, ‘bedding materials’, ‘litter management’). Subsequently, the full papers have been retrieved through the websites of the respective journals. Citations of other materials are scarce; these include a few references to book chapters and conference material. After a search that has covered about 150 manuscripts, references cited appear at the end of the review.

## 3. Dairy Farm Environment 

Dairy farms have a dynamic environment with associated microbial communities, which have natural reservoirs among components of this ecosystem [12]. This environment is very complex due to the microbial diversity present in the different areas of the farm (such as the milking parlor, the stables, and even the environment surrounding the farm), and it does not guarantee good application of the Hazard Analysis Critical Control Points system. For this reason, the health authorities recommend the application of good manufacturing practices on the farm, as the European legislation has done (Regulation EC n° 178/2002 [13] and Regulation EC n° 853/2004 [4]).

Adequate sanitary environmental conditions in food processing plants could reduce the level of the microbial communities on the farm. However, it is impossible to keep environmental contamination, such as bacteria, yeasts, and molds, at a minimum level in the environment of food processing [14]. This is mainly due to the air present in the environment, which is the transport vehicle of matter particles and bioaerosols. For this reason, it is important to pay special attention to the quality of this air, as one of the main routes of contamination of dairy farms. The air is a hostile environment as a habitat for microorganisms because they are stressed by the lack of nutrients and dehydration, but it is an important vehicle for dissemination since we can find all kinds of bacteria in it [15]. The microorganisms could travel through the air adhered to dust particles or droplets, or even as single particles, until they fall and are deposited on the surrounding surfaces [16]. The air, as a source of contamination through dissemination, has been properly researched in numerous studies of food processing plants [14] as well as in the field of enology [17].

Several studies related to environmental contamination on dairy farms have focused on pathogens and decomposition bacteria. As far as is known, few studies have focused on environmental microbiology, from the point of view of contamination and its effect on milk and dairy products [18]. It is now clear that the microbiological load present in the environment of farms could have a positive influence on milk, inasmuch as the possible existence of some airborne microorganisms, such as lactic acid bacteria, would permit an enrichment of the nutritional and organoleptic qualities of the milk, as has been previously studied in wineries [17]. In addition, the microbiota naturally present in milk may have a protective effect against the growth of pathogens found in the air [19]. However, the ability of the airborne microbial community to contaminate milk remains an ongoing concern, and the sources of the origin of contamination of those microorganisms that might affect the natural microbiota of milk have not been studied in depth in dairy cattle [18], and not studied at all in sheep or goats. Thus, limited information is available on the microbiological quality of indoor air on dairy farms and the factors that influence it [20], where the management of different substrates used to feed the animals and change the bedding, along with herd size and udder hygiene, may contribute to the transfer between the microorganisms present in the environment and the milk.

As regards the milking practice, which depends on the hygienic practices of the farmer during the process, the environmental sources of contamination may play an important role in the transfer of microorganisms, but this issue has not yet been carefully examined [18]. During and after milking, microbial contamination may occur through the different environmental sources, such as feeds, feces, bedding material, and soil, which adhere to the udder and from there, are transferred to the milk [21]. In view of the diversity of microbiota, we cannot discard the hypothesis that certain microorganisms present in the environment could be later found in the raw milk because of the animals housed in the stables. This transfer from the environment to the milk could take place either directly by air or through the teat, which has been in contact with various media (litter, dust, etc.), or because of the milking practice.

As previously mentioned, housing hygiene could have great importance in the control of dairy farm contamination, because it is considered an important source of microorganisms that could affect the quality of the milk [5]. Vissers et al. [22] found that the level of spore-forming butyric acid bacteria contamination was higher in cows that lay down on dirty patches of the housing, which resulted in highly contaminated teats before milking. This teat hygiene is related to the amount of dirt transmitted to the milk by a factor of 100 difference between a well-managed farm and badly-managed farm [23]. In addition, the hygiene of livestock can have an influence on the transmission of diseases. Rolesu et al. [24] showed that correct hygiene, with continuous removal of mud and manure from the livestock housing, drastically reduces the possibility of any outbreak of disease that could affect the animals.

As is the case with the dairy sector, environmental contamination in the cheese industry is of vital importance in the cheese-making process. Air is an important source of contamination, with microorganisms being transported through it, affecting the properties of the final product [25]. In addition, the hygienic quality of milk is very important, especially for milk that will be used for the elaboration of unpasteurized cheese [26]. For this reason, it is important to pay attention to possible routes of contamination through the dairy farm environment.

## 4. Effect of Temperature on Air Quality

Temperature plays an important role in the environment of dairy farms because it is closely related to the welfare of the animals. In the coming years, due to climate change, the average global temperatures could rise by up to 4 °C in some parts of the world [27]. This increase will have an important impact on livestock farming systems, as well as on human and animal health [28].

The effect of temperature on dairy farms depends on both the location and the season. In general, the different microclimatic conditions in each locality play a crucial role in the microbiological population, given that these microbial communities are very complex and variable to seasonal changes [29]. In this context, some researchers observed differences in the concentration of microorganisms in the environment, depending on the season [30] or due to meteorological factors [31]. As Sanz et al. [15] observed in their research of a dairy cattle farm, there was a seasonal effect on the number of isolates of bacteria (*Escherichia coli*) in the air, with up to twice the amount in the hot season (summer) than in the cold season (winter). Even the time of day influences the concentration of microorganisms. Popescu et al. [20] found a positive correlation between the increase in temperature and the increase in the mesophilic bacteria count in the environment, both in the morning and in the evening. For this reason, it is clear that seasonal and daily variations exist that make the bacterial counts higher in the hottest seasons as well as in the hottest hours of the day.

Environmental microorganisms are positively correlated with air temperature, but negatively correlated with humidity and solar radiation [31]. Regarding this fact, [32], in his research, concluded that if the study had been carried out in summer, the number of microorganisms detected in the air would probably have been higher. Calamari et al. [33], according to their research, noted a greater content of spores in feces during summer. These spores colonize the skin and the hair of animals and can contaminate the dairy farm environment. However, Adhikari et al. [34] found a higher concentration of spores and microorganisms in the environment in winter, indicating that it may be due to microclimatic differences between countries in which studies of this type are carried out, or that differences in diet composition might interfere with seasonal effects.

Good building design provides protection against climatic conditions (especially in areas which are more susceptible to extreme weather conditions), alleviating stress in the animals and avoiding increases in respiration rate and exchanges between the animal’s body surface and the environment, which contribute to air pollution [35]. The choice of the adequate interval of temperature depends on the farming system. In dairy cattle, it is important to keep the animals between −5 °C and 22 °C [36], although this thermal comfort zone varies depending on the physical condition of the animal, the resources available, and environmental factors. In the case of small ruminants, the optimum air temperature for efficient production should range between 5 °C and 20 °C [37].

In terms of animal welfare, some measures are strictly recommended depending on the season. During summer, sheep exposed to high temperatures and solar radiation show an increase in their respiration rate, an increase in their rectal temperature, and a metabolic alteration as opposed to those that stay in the shade (influencing the air quality), so the milk coagulating properties are diminished [38]. In addition, exposure to such radiation reduces the defensive capacity of the udder, which may allow contamination of the mammary gland by environmental pathogens [35]. Hence, providing shade in the barn during the hours of extreme heat minimizes the impact on the animal´s body surface, improving the quality of the air and, thus, the quality of the milk [37]. During winter, the thermal comfort zone may vary depending on the physical and health conditions of the animal, the feeding, and other environmental factors. Therefore, the minimum thermal comfort value could be increased when the animals are wet or exposed to airflows or decreased when the relative humidity and solar radiation are high [39].

Airborne microbiota is also affected by the activities of the farm, and this could affect the environmental analysis developed. The best moment of the day to collect a sample or to measure certain parameters would be during the morning, before the aeration of the farm and before the beginning of farming activities, which contaminate the environment [20]. In addition, carrying out these analyses in the morning avoids the bactericidal effect of the radiation and helps to collect a wide range of airborne microorganisms [40].

The climate conditions also have an effect on the milking equipment. Poor hygiene in the milking parlor contributes to the pollution of the dairy farm environment and results in the contamination of the milking equipment. This situation, combined with high temperatures in the period between milking, increases the growth rate of microorganisms that affect the level of contamination in the milk [21]. This aspect is important when the milk is destined for use in the cheese-making process because the quality of the final product may be compromised.

## 5. Effect of Humidity on Air Quality

A good livestock housing design is a fundamental factor to avoid the proliferation of microorganisms in the environment of dairy farms, and an essential aspect to be considered for the optimum project is humidity.

Relative humidity is important for health because it is one of the main factors that affect respiratory damage in both humans and animals. As Xiong et al. [41] said, the level of humidity is important for the welfare of animals because the infectivity of pathogens found in the environment depends on this level.

It is important to take into account that the main parameters on the farm are interrelated: without good air distribution, poor ventilation will occur, the temperature will fluctuate from its optimum range, and relative humidity will be affected, influencing the count of microorganisms and molds. Moreover, as all environmental parameters are related in dairy farms, Sevi et al. [42] and Jimeno [43] concluded that certain space in the stables is necessary for each animal to ensure the correct relative humidity. If that living space is diminished, the concentration of pathogenic microorganisms in the environment is increased.

Dairy animals tolerate moisture poorly. The recommended values of relative humidity in dairy farms are between 55% and 75% for dairy cattle [36] and ≤70% for small ruminants [37]. However, these values overlap with the optimal level for the survival of most bacteria and fungi 55%–75% [41]. Moreover, Wilson et al. [40] found that low humidity negatively affects the collection of microorganisms from the environment, which may be due to their lower presence. For this reason, control of the humidity is necessary to minimize the risk.

Although Tang [44] found that the relationship between airborne bacteria with relative humidity is complicated, some researchers discovered that a positive correlation exists between the increased humidity of the air and fungi [20]. This indicates that fungi are the ones most affected by humidity. This is due to the fact that humid conditions induce the decomposition of organic materials in the livestock housing, which provides an excellent setting for the growth of fungi, and this contributes to an increase in the spore load in the farm environment [34]. In addition, it can be stated that there is a correlation between the concentrations of fungi in the environment and the different values of the relative humidity of indoor surfaces [45]. Therefore, it seems that there is a positive correlation between the humidity level of livestock housing and the fungi load in the environment.

## 6. Effect of Livestock Housing on Air Quality

The livestock housing has an important role in the dairy farm environment because a good design that promotes good health and welfare for the different species is a vital aspect of sustainable animal production, along with proper hygiene. The hygiene in the animal sheds of the farms includes air, bedding, and surface hygiene. Poor hygiene of surfaces is connected to intensive farming systems, and is aggravated by poor maintenance [46].

Microbial communities present in livestock housing are interrelated with those present in the milking parlor. Good milking practice is another factor in a proper dairy farm environment because the correct management of the milking parlor improves the quality of the milk [18]. Hence, good hygiene among the farm-workers during milking (cleaning of the machines, hygienic parlor, udder disinfection, etc.) affects the concentration of microorganisms in the environment, both those bacteria which are favorable to the quality of the milk and those which are harmful to this quality [47]. Generally, increasing the times between two milking intervals provides the microorganism with more time to grow, increasing the microbial concentration in the environment that surrounds the milking equipment, and affecting the level of contamination in the milk [21]. In addition, poor hygiene has negative effects on milk yield, causing low concentrations of protein and fat; and an increase in somatic cells in the animals of the herd, which in turn decreases the quality of the milk [9].

It is required that the dairy farm be well designed. Proper air space for the animals is one of the most important factors for the quality of the air and the welfare of the animals in the livestock houses. Air quality in the livestock building is a source of contamination not only for the animals but also for the workers’ health. The optimal volume to avoid an increase in the total mesophilic bacteria count and the relative humidity in the environment, as well as a decrease in the properties of the milk, is 35–40 m^3^/head for cattle [48] and 7 m^3^/head for sheep and goats [38].

Another factor that affects air quality in livestock is the stocking density. The concentration of microorganisms and particles in the environment in livestock housing is inversely related to space. Concentrations of total mesophilic bacteria in the livestock house is significantly lower when animals have a stocked density of 5 m^2^/head for cows [43] and 2 m^2^/head for sheep or goats, and the somatic cell count in small ruminants is up to four times lower than animals stocked at 1–1.5 m^2^/head [42].

Furthermore, the material used in livestock housing affects the concentration of microorganisms in the air. If there is hay on the floor of the housing, it may increase the propionic bacteria and *lactobacillus* [49]; the use of sand increases the number of *Streptococcus,* and the use of sawdust increases the level of coliforms and *Klebsiella* in the animals [50]. However, Monsallier et al. [51] showed that the use of straw as a bedding material, as opposed to other materials, means an increase in the microbial levels of the herds, which implies a higher level of ripening bacteria in the milk.

The material that covers the floor of the house is a source of contamination. The concentration of microorganisms in the dairy farm environment tends to be higher in those farms where the bedding is not clean or is not changed frequently [20,52]. Therefore, it is necessary to change the bedding material daily or add another layer of material each day to reduce the microbial load significantly. It has been demonstrated that fresh bedding usually has much lower microbial concentrations than used bedding because the concentration of microorganisms tends to increase due to contamination from feces and microbial growth during the first day [53]. Treating the bedding with products that reduce microbial activity (such as bentonite) can be a good strategy to reduce contamination in the livestock environment [46]. Bentonite is a volcanic material composed mainly of clay minerals and has a great capacity for water absorption. The application of bentonite to the bedding achieves a significant reduction in the number of microorganisms in the environment, which improves the quality of the milk [52]. It can also partially alleviate the adverse effects of the stocking density in farms, improving the coagulation capacity and, thus, the quality of the milk [54]. Therefore, good maintenance of bedding on farms is a critical point for livestock hygiene. It is clear that there is a transfer of microorganisms between the environment and the milk, and it is for this reason that good health and hygienic parameters are very important in the production of high-quality milk.

Apart from those factors already mentioned, another significant parameter is the feed. Diet may play an important role in the dairy farm environment because the air quality is influenced by the feces of the animals, which is an important contamination factor. It is well known that feed introduces a large variety of microorganisms and spores into the environment and, therefore, into the milk [21]. This can be seen in the results of Calamari et al. [33], where the concentration of spores in feces, and consequently, the contamination of the environment, is strictly related to the content of spores in the feed. It is important to control the impact that the feed has on the dairy farm environment because it not only affects the quality of the milk but also acts as a disseminator of pathogens affecting the animals on the farm.

Despite the high number of microorganisms present in the air in the dairy farm environment, only a third of them are detected in the milk [18], which means that there is a partially efficient (but not impervious) barrier between the barn and the milk. The effectiveness of this barrier is influenced by the hygienic conditions of the farm. The better the hygiene in livestock house, the lower the count of total mesophilic bacteria and the higher the quality of the milk. In this regard, Oliete et al. [55] demonstrated with ovine that the quality of the milk is related to good hygienic practices on the farm. In a similar case studied by Bergonier et al. [56], a relation was found between the poor hygiene of the milking parlor and the livestock house and the increase in total mesophilic bacteria, and, thus, poor milk quality.

As previously mentioned, the environment of livestock housing has important effects on the quality of milk. This implies that the quality of dairy products, particularly in the cheese-making industry, could be affected too. It is essential to consider the correct parameters in animal welfare, which have an influence on dairy products. As Sevi et al. [42] demonstrated, the milk from ewes stocked at 2 m^2^/head showed a significant increase in milk coagulating properties, avoiding harmful bacteria in the environment and enhancing cheese-making bacteria crucial for the manufacturing process. In addition, Albenzio et al. [57] discovered that the control of housing sanitation and milking procedures is critical to obtain good quality milk and its products. Meanwhile, Monsallier et al. [51] demonstrated the importance of bedding material as a source of beneficial bacteria in ripening for cheese making, with the type of bedding material used for the stables acquiring major significance.

## 7. Effect of Ventilation on Air Quality

The different ventilation regimens in animal houses have the main objective of maintaining optimum air quality to control environmental contamination. Certain environmental pathogens may be under control to obtain an acceptable quality of the air that the animals breathe [58]. For this reason, a proper ventilation regimen should provide the necessary oxygen for animals, eliminate harmful gases, eliminate excess water vapor, decrease the temperature of the animal houses, eliminate dust and odors, and decrease the concentration of microorganisms. It is important to note that the magnitude of these objectives, in each particular case, depends on the climate, the time of year, the age of the animals, and other factors, such as the surrounding buildings or the breeding shed orientation. Orientation is one of the most influential factors, mainly due to greater or lesser exposure to wind and solar radiation, which affect the exchange of air between the exterior and the interior of the building [39].

On dairy farms, the best ventilation system in animal houses is natural ventilation, which occurs due to the natural phenomena of pressure and differences of temperature between different spaces and between indoor/outdoor farm locations. However, a dynamic ventilation system can sometimes be used, where the air is introduced into or extracted from the livestock houses by fans with a fixed flow rate, although this is not the most suitable system for dairy farms [58]. In the case of dairy farms, it is preferable to use the natural ventilation systems, and even better to keep animals in outdoor enclosures during the day, because it is beneficial for the air quality and animal welfare and, consequently, for the quality of the milk [59].

It has been demonstrated that the wind and its speed, have a great influence on the transport of microorganisms. There are some research projects which focus on the dispersion of bacteria due to wind in different environments [17,20], while other authors describe how the wind influences the dissemination of fungi [60].

The livestock houses are the basis of the health of the farmed animals and must act as the respiratory system of the farm. Ventilation rates and the quality of air delivery and exchange in animal houses are significant factors in animal health and disease protection [61] because adequate ventilation will prevent the accumulation of pathogens, airborne contaminants, and dust inside the building, thus avoiding the transmission of infections in the livestock and their environment. Poor ventilation is responsible for an increase in environmental pollution and exchanges between the animal’s body surface and the environment in the livestock houses [35]. In this sense, it is very important to maintain adequate ventilation rates on the farms, due to the contamination present in this environment. The microbial contamination in the air sometimes reaches concentrations higher than 10^4^ ufc/m^3^ [62], and Sevi et al. [37] recommend less than 250 ufc/m^3^ to improve animal welfare. In addition, ventilation is essential to maintain the health and production of dairy cattle [63], so it plays a very important role in animal welfare, since it prevents an excessive increase in relative humidity and keeps the levels of harmful gases, dust and particles from the environment under control [64].

As previously mentioned, an important factor that significantly affects the microbial load is the ventilation rate of the installations. There are numerous studies that recommend ventilation rates for each type of farm, such as cattle [65] or sheep [26]. In general, these studies show that a low ventilation regime accompanied by few ventilation cycles results in a lower hygienic–sanitary quality of the milk.

Seasonality is a factor to take into account in the air quality due to the ventilation rates of livestock. During summer, it is necessary to have an average ventilation of around 1700 m^3^/h per head in dairy cattle [58] and around 65 m^3^/h per head in dairy sheep or goats [66]. However, in winter, it is better to have a moderate ventilation rate of around 85 m^3^/h per head for cattle [58] and around 45 m^3^/h per head for sheep or goats [37]. The importance of ventilation during winter is often underestimated [67]. It has been observed that poor ventilation results in an increase in gasses from the respiratory activity of the animals and an increase in humidity; however, high ventilation results in a high concentration of dust, probably due to a turbulent air regime caused by the high rate of ventilation that keeps the particles suspended in the air for a long time. Inadequate ventilation regimes deteriorate milk quality, and higher levels of somatic cell and mesophilic bacteria are counted [68]. Moreover, it is advisable to use a fan speed of 0.5 m/s in the case of dairy cattle [36], and 2 m/s in the case of small ruminants to sustain the air quality [26,67], and, therefore, improve the quality of the milk.

An increase in the rate of ventilation between the different areas of the farm is negatively correlated with the concentration of microorganisms in the environment. For this reason, the location of the entrances and exits of air in the areas where the animals are resting has a great influence on the concentration and propagation of the microbial contamination of indoor air, as Gustaffson explained [69]. The effects of stocking density are also important in air quality. Hartung [70] claimed that the less volume of air the animals have, the more an appropriate ventilation system is needed in the animal housing.

The wind and the rate of ventilation on the farm is a source of pollution for the dairy industry and its products because it is a determining factor in the dispersion of bacteria [15]. For that reason, the cheese industry may also be affected by poor ventilation on the dairy farm. It is demonstrated that wind is a factor to be taken into account in the cheese-making process, because the renneting properties of milk may be improved by increasing the ventilation rate to 70 m^3^/h or by increasing the length of ventilation cycles to 60 min in goat farms [26]. In addition, the ventilation rate per head could improve the quality of the milk and, consequently, the cheese-making process.

Adequate preventive measures in the dairy farm environment are the best guarantee to ameliorate the welfare of the animals and to control the influence that the microbial communities have on indoor air quality. Poor air quality on the farm could affect milk hygiene, so there could be important economic and health consequences for farmers, animals, and consumers. For this reason, it is important to establish a series of recommendations to control the parameters in the dairy farm environment, with the objective of maintaining the correct air quality (Table 1).

## 8. Conclusions

Two interrelated factors, such as temperature and humidity, influence environmental contamination and animal welfare, and this effect is more pronounced where the climatology is extreme. The proper choice of a temperature and humidity range for the animals avoids a higher concentration of microorganisms in the dairy environment as well as an exchange of microorganisms between the environment and the animals housed. In areas with extreme weather, several precautions must be taken in the farm design to maintain animal welfare.

Nowadays, it is well known that the design of buildings on a farm is a critical point to avoid pathologies in the animals housed on it because these pathogens could appear in a contaminated environment. The concentration of microorganisms in the environment depends on the space available to the livestock, so it is necessary to establish a series of acceptable values that allow keeping the animals in healthy conditions. In addition, the proper selection of bedding material and the frequent changing of this material helps to maintain the air quality.

Similarly, the environmental conditions in the animals’ housing and the milking parlor are some of the main criteria in the design of a farm. Depending on the weather, it is necessary to provide the correct ventilation rate for each type of dairy livestock, as well as the appropriate air velocity within the spaces. Combined with the control of dangerous gases (NH_3_, CO_2_, and H_2_S), dust and particles, these parameters ensure a better air quality on the dairy farm and, thus, improved wellbeing among the livestock.

Taking all the above into consideration, it is necessary to study the dairy farm environment in-depth to investigate the influence that microorganisms have on the health of animals depending on environmental conditions, as well as to establish preventive measures to avoid economic and health consequences.

## Figures and Tables

**Table 1:** Recommendations in some parameters of the dairy farm environment to proper sanitary environmental conditions and, consequently, improve the quality of the final product.

Air Quality Recommendations	Cow Dairy Farm	Sheep Dairy Farm	Goat Dairy Farm
Ventilation Rate (Winter-Summer)	85–1700 m^3^/h/head [58]	47–65 m^3^/h/head [66,67]	
Stocking density	5 m^2^/head [43]	2 m^2^/head [42]	
Air space	35–40 m^3^/head [48]	7 m^3^/head [38,71]	
Air velocity	<0.5 m/s [36]	2 m/s [26,66]	
Humidity	55–75% [36]	≤70% [37]	
Tª (min-max)	−5 °C–22 °C [36]	5 °C–20 °C [37]	
Dust	1.7–3.4 mg/m^3^ air [46]	<1.6 mg/m^3^ air [37]	
NH_3_	<20 ppm [46]	<10 ppm [37,64]	
CO_2_	<3000 ppm [46]	<2500 ppm [37,64]	
H_2_S	<0.5 ppm [46]	<2.5 ppm [37,64]	
Bedding material	Straw [51]		

## References

[1] FAO Food Outlook. http://www.fao.org.

[2] Kapaj A., Deci E., Watson R.R., Collier R.J., Preedy V.R. (2017). World Milk Production and Socio-Economic Factors Effecting Its Consumption. Dairy in Human Health and Disease Across the Lifespan.

[3] Arias R., Jiménez L., Oliete B., Murillo G., García A., Lara M., Plaza L., Rodríguez D. (2015). Calidad y seguridad de la leche en la producción primaria. Programas de autocontrol en las ganaderías. Gestión Sustentable de Empresas Agroalimentarias. Factores Clave de Estrategias Competitivas.

[4] Regulation E.C. (2004). Regulation (EC) No 853/2004 of the European Parliament and of the Council of 29 April 2004 laying down specific hygiene rules for food of animal origin. Off. J. Eur. Union.

[5] Jiménez L., Oliete B., Pérez-Guzmán M.D., Arias R. Influencia de la estación, tamaño de explotación y asociacionismo en el recuento de microorganismos en leche de tanque de oveja. Proceedings of the XVI Jornadas sobre Producción Animal AIDA.

[6] Gonzalo C., Carriedo J.A., Beneitez E., Juárez M.T., De La Fuente L.F., San Primitivo F. (2006). Short communication: Bulk tank total bacterial count in dairy sheep: Factors of variation and relationship with somatic cell count. J. Dairy Sci..

[7] Quigley L., O’Sullivan O., Beresford T.P., Ross R.P., Fitzgerald G.F., Cotter P.D. (2011). Molecular Approaches to Analysing the Microbial Composition of Raw Milk and Raw Milk Cheese. Int. J. Food Microbiol..

[8] Kyozaire J.K., Veary C.M., Petzer I.M., Donkin E.F. (2005). Microbiological quality of goat‘s milk obtained under different production systems. J. S. Afr. Vet. Assoc..

[9] Arias C., Oliete B., Seseña S., Jiménez L., Pérez-Guzmán M., Arias R. (2013). Importance of on-farm management practices on lactate-fermenting Clostridium spp. spore contamination of Manchega ewe milk: Determination of risk factors and characterization of Clostridium population. Small Rumin. Res..

[10] Quigley L., O’Sullivan O., Stanton C., Beresford T.P., Ross R.P., Fitzgerald G.F., Cotter P.D. (2013). The complex microbiota of raw milk. FEMS Microbiol. Rev..

[11] Fischer W.J., Schilter B., Tritscher A.M., Stadler R.H. (2015). Contaminants of Milk and Dairy Products: Contamination Resulting from Farm and Dairy Practices. Ref. Modul. Food Sci..

[12] McAuley C.M., McMillan K., Moore S.C., Fegan N., Fox E.M. (2014). Prevalence and characterization of foodborne pathogens from Australian dairy farm environments. J. Dairy Sci..

[13] (2002). Regulation (EC) No 178/2002 of the European Parliament and of the Council of 28 January 2002 laying down the general principles and requirements of food law, establishing the European Food Safety Authority and laying down procedures in matters of food safety. Off. J. Eur. Comm..

[14] Brandl H., Fricker-Feer C., Ziegler D., Mandal J., Stephan R., Lehner A. (2014). Distribution and identification of culturable airborne microorganisms in a Swiss milk processing facility. J. Dairy Sci..

[15] Sanz S., Olarte C., Martínez-Olarte R., Navajas-Benito E.V., Alonso C.A., Hidalgo-Sanz S., Somalo S., Torres C. (2015). Airborne dissemination of Escherichia coli in a dairy cattle farm and its environment. Int. J. Food Microbiol..

[16] Curiel G.J., Van Eijk H.M.J., Lelieveld H.L.M., Robinson R.K., Batt C.A., Patel P.D. (2000). Risk and control of airborne contamination. Encyclopedia of Food Microbiology.

[17] Perez-Martín F., Seseña S., Fernandez-Gonzalez M., Arevalo M., Palop M.L. (2014). Microbial communities in air and wine of a winery at two consecutive vintages. Int. J. Food Microbiol..

[18] Vacheyrou M., Normand A.C., Guyot P., Cassagne C., Piarroux R., Bouton Y. (2011). Cultivable microbial communities in raw cow milk and potential transfers from stables of sixteen French farms. Int. J. Food Microbiol..

[19] Donnelly C.W. (2001). Factors associated with hygienic control and quality of cheeses prepared from raw-milk: A review. Bull. Int. Dairy Fed..

[20] Popescu S., Borda C., Diugan E.A. (2011). Microbiological air quality in tie-stall dairy barns and some factors that influence it. Afr. J. Agric. Res..

[21] Vissers M.M.M., Driehuis F., Tamime A.Y. (2009). On-farm hygienic milk production. Milk Processing and Quality Management.

[22] Vissers M.M.M., Driehuis F., Te Giffel M.C., De Jong P., Lankveld J.M.G. (2006). Improving farm management by modeling the contamination of farm tank milk with butyric acid bacteria. J. Dairy Sci..

[23] Vissers M.M.M., Driehuis F., Te Giffel M.C., De Jong P., Lankveld J.M.G. (2007). Short communication: Quantification of the transmission of microorganisms to milk via dirt attached to the exterior of teats. J. Dairy Sci..

[24] Rolesu S., Loi F., Cappai S., Coccollone A., Cataldi M., Usala P., Podda A., Deliperi S., Oppia P., Natale A. (2018). Description and typology of dairy sheep farm management profiles in Sardinia. Small Rumin. Res..

[25] Kure C.F., Skaar I., Brendehaug J. (2004). Mould contamination in production of semi-hard cheese. Int. J. Food Microbiol..

[26] Albenzio M., Santillo A., Caroprese M., Marino R., Centoducati P., Sevi A. (2005). Effect of different ventilation regimens on ewes’ milk and Canestrato Pugliese cheese quality in summer. J. Dairy Res..

[27] Yatoo M.I., Kumar P., Dimri U., Sharma M.C. (2012). Effects of climate change on animal health and diseases. Int. J. Livest. Res..

[28] Marino R., Atzori A.S., D’Andrea M., Iovane G., Trabalza-Marinucci M., Rinaldi L. (2016). Climate change: Production performance, health issues, greenhouse gas emissions and mitigation strategies in sheep and goat farming. Small Rumin. Res..

[29] Pangloli P., Dje Y., Ahmed O., Doane C.A., Oliver S.P., Draughon F.A. (2008). Seasonal incidence and molecular characterization of Salmonella from dairy cows, calves, and farm environment. Foodborne Pathog. Dis..

[30] Vissers M.M.M., Te Giffel M.C., Driehuis F., De Jong P., Lankveld J.M.G. (2007). Minimizing the level of Bacillus cereus spores in farm tank milk. J. Dairy Sci..

[31] Dungan R.S., Leytem A.B., Bjorneberg D.L. (2011). Concentrations of airborne endotoxin and microorganisms at a 10,000-cow open-freestall dairy. J. Anim. Sci..

[32] Karwowska E. (2005). Microbiological air contamination in farming environment. Pol. J. Environ. Stud..

[33] Calamari L., Morera P., Bani P., Minuti A., Basiricò L., Vitali A., Bernabucci U. (2018). Effect of hot season on blood parameters, fecal fermentative parameters, and occurrence of Clostridium tyrobutyricum spores in feces of lactating dairy cows. J. Dairy Sci..

[34] Adhikari A., Sen M.M., Gupta-Bhattacharya S., Chanda S. (2004). Volumetric assessment of airborne fungi in two sections of a rural indoor dairy cattle shed. Environ. Int..

[35] Caroprese M. (2008). Sheep housing and welfare. Small Rumin. Res..

[36] Bureau B.T.P.L. (2001). Technique de Promotion Laitière.

[37] Sevi A., Casamassima D.V., Pulina G., Pazzona A. (2009). Factors of welfare reduction in dairy sheep and goats. Ital. J. Anim. Sci..

[38] Sevi A., Annicchiarico G., Albenzio M., Taibi L., Muscio A., Dell’Aquila S. (2001). Effects of solar radiation and feeding time on behavior, immune response and production of lactating ewes under high ambient temperature. J. Dairy Sci..

[39] Núñez N., Callejo A. (2006). Condiciones ambientales y bienestar. La ventilación. Frisona Española.

[40] Wilson S.C., Morrow-Tesch J., Straus D.C., Cooley J.D., Wong W.C., Mitlöhner F.M., McGlone J.J. (2002). Airborne microbial flora in a cattle feedlot. Appl. Environ. Microbiol..

[41] Xiong Y., Meng Q.S., Gao J., Tang X.F., Zhang H.F. (2017). Effects of relative humidity on animal health and welfare. J. Integr. Agric..

[42] Sevi A., Massa S., Annicchiarico G., Aquila S.D., Muscio A. (1999). Effect of stocking density on ewes’ milk yield, udder health and microenvironment. J. Dairy Res..

[43] Jimeno V. (2004). Diseño de alojamientos para vacas lecheras en estabulación libre. Curso Para Peritos Tasadores.

[44] Tang J.W. (2009). The effect of environmental parameters on the survival of airborne infectious agents. J. R. Soc. Interface.

[45] Pasanen A.L., Rautiala S., Kasanen J.P., Raunio P., Rantamäki J., Kalliokoski P. (2000). The relationship between measured moisture conditions and fungal concentration in water-damaged building materials. Indoor Air.

[46] Wathes C.M., Wathes C.M., Charles D.R. (1994). Air and surface hygiene. Livestock Housing.

[47] Verdier-Metz I., Michel V., Delbès C., Montel M.C. (2009). Do milking practices influence the bacterial diversity of raw milk?. Food Microbiol..

[48] Callejo A., Buxadé C. (1998). Alojamiento para vacas lecheras. Alojamientos e Instalaciones (II).

[49] Bouton Y., Guyot P., Vacheyrou M., Normand A.C., Piarroux R., Beuvier E. Etude des flux bactériens dans les étables de production laitière de Franche-Comté. Exemple des LHF. Proceedings of the 15ème colloque du Club des Bactéries Lactiques.

[50] Zdanowicz M., Shelford J.A., Tucker C.B., Weary D.M., von Keyserlingk M.A. (2004). Bacterial populations on teat ends of dairy cows housed in free stalls and bedded with either sand or sawdust. J. Dairy Sci..

[51] Monsallier F., Feutry F., Bouton Y., Convert T., Verdier-Metz I., Montel M.C. Are bedding materials a source of useful microorganisms for dairy cow and ewe milk?. Proceedings of the Joint Meeting FAO CIHEAM “Mountain Pastures, Mediterranean Forage Resources and Mountain Cheese” networks.

[52] Sevi A., Massa S., Muscio A., Dell’aquila S.D.D., Catalano S. (1998). Litter treatment with bentonite or paraformaldehyde: Effects on air quality and on milk yield of Comisana ewes. Zootec. Nutr. Anim..

[53] Hogan J.S., Bogacz V.L., Thompson L.M., Romig S., Schoenberger P.S., Weiss W.P., Smith K.L. (1999). Bacterial counts associated with sawdust and recycled manure bedding treated with commercial conditioners. J. Dairy Sci..

[54] Sevi A., Taibi L., Muscio A., Albenzio M., Dantone D., Dell’Aquila S. (2001). Quality of ewe milk as affected by stocking density and litter treatment with bentonite. Ital. J. Food Sci..

[55] Oliete B., Calatayud M., García O., Arias C., Gallego R., Arias R., Pérez-Guzmán M.D. (2010). Efecto de las condiciones higiénico-sanitarias sobre el recuento de células somáticas y microorganismos totales de la leche de oveja Manchega. XXXV Congreso de la Sociedad Española de Ovinotecnia y Caprinotecnia.

[56] Bergonier D., De Crémoux R., Rupp R., Lagriffoul G., Berthelot X. (2003). Mastitis of dairy small ruminants. Vet. Res..

[57] Albenzio M., Caroprese M., Marino R., Muscio A., Santillo A., Sevi A. (2006). Characteristics of Garganica goat milk and Cacioricotta cheese. Small Rumin. Res..

[58] Callejo A. (2013). Ventilación de alojamientos de vacas de leche. Funciones y diseño. Frisona Española 197.

[59] Casamassima D., Sevi A., Palazzo M., Ramacciato R., Colella G.E., Bellitti A. (2001). Effects of two different housing systems on behaviour, physiology and milk yield of Comisana ewes. Small Rumin. Res..

[60] Sen B., Asan A. (2009). Fungal flora in indoor and outdoor air of different houses in Tekirdag City (Turkey): Seasonal distribution and relationship with climatic factors. Environ. Monit. Assess..

[61] Murphy D., Ricci A., Auce Z., Beechinor J.G., Bergendahl H., Breathnach R., Bureš J., Duarte Da Silva J.P., Hederová J., Hekman P. (2017). EMA and EFSA Joint Scientific Opinion on measures to reduce the need to use antimicrobial agents in animal husbandry in the European Union, and the resulting impacts on food safety (RONAFA). EFSA J..

[62] Bouton Y., Laithier C. (2011). L’environnement des animaux. Microflore du Lait cru. Vers Une Meilleure Connaissance des Écosystèmes Microbiens du Lait de Leurs Facteurs de Variation.

[63] Lange J.L., Thorne P.S., Kullman G.J. (1997). Determinants of Culturable Bioaerosol Concentrations in Dairy Barns. Ann. Agric. Environ. Med..

[64] Sevi A., Gabina D., Le Jaouen J.C., Pirisi A., Ayerbe A., Soustre Y. (2005). Influence of sunlight, temperature and environment on the fatty acid composition and coagulatine properties of sheep milk. The Future of the Sheep and Goat Dairy Sectors.

[65] Lawrence N.G., Wathes C.M., Charles D.R. (1994). Beef cattle housing. Livestock Housing.

[66] Sevi A., Taibi L., Albenzio M., Annicchiarico G., Marino R., Caroprese M. (2003). Influence of ventilation regimen on microenvironment and on ewe welfare and milk yield in summer. Ital. J. Anim. Sci..

[67] Sevi A., Taibi L., Albenzio M., Caroprese M., Marino R., Muscio A. (2003). Ventilation effects on air quality and on the yield and quality of ewe milk in winter. J. Dairy Sci..

[68] Albenzio M., Marino R., Caroprese M., Santillo A., Annicchiarico G., Sevi A. (2004). Quality of milk and of Canestrato pugliese cheese from ewes exposed to different ventilation regimens. J. Dairy Res..

[69] Gustafsson G. (1997). Investigations of factors Affecting Air Pollutants in Animal Houses. Ann. Agric. Environ. Med..

[70] Hartung J., Wathes C.M., Charles D.R. (1994). Environment and animal health. Livestock Housing.

[71] Sevi A., Taibi L., Albenzio M., Annicchiarico G., Muscio A. (2001). Airspace effects on the yield and quality of ewe milk. J. Dairy Sci..

